# β-galactosidase Production by *Aspergillus niger* ATCC 9142 Using Inexpensive Substrates in Solid-State Fermentation: Optimization by Orthogonal Arrays Design

**DOI:** 10.22045/ibj.2016.06

**Published:** 2016-11

**Authors:** Samaneh Kazemi, Gholam Khayati, Mohammad Faezi-Ghasemi

**Affiliations:** 1 Vice-Chancellor of Research and Technology, Guilan University of Medical Sciences, Rasht, Iran; 2 Department of Chemical Engineering, Faculty of Engineering, University of Guilan, Rasht, Iran; 3 Department of Microbiology, Faculty of Science, Islamic Azad University, Lahijan Branch, Lahijan, Iran

**Keywords:** Beta-galactosidase, *Aspergillus niger*, Solid waste

## Abstract

**Background:**

Enzymatic hydrolysis of lactose is one of the most important biotechnological processes in the food industry, which is accomplished by enzyme β-galactosidase (β-gal, β-D-galactoside galactohydrolase, EC 3.2.1.23), trivial called lactase. Orthogonal arrays design is an appropriate option for the optimization of biotechnological processes for the production of microbial enzymes.

**Methods:**

Design of experimental (DOE) methodology using Taguchi orthogonal array (OA) was employed to screen the most significant levels of parameters, including the solid substrates (wheat straw, rice straw, and peanut pod), the carbon/nitrogen (C/N) ratios, the incubation time, and the inducer. The level of β-gal production was measured by a photometric enzyme activity assay using the artificial substrate ortho-Nitrophenyl-β-D-galactopyranoside.

**Results:**

The results showed that C/N ratio (0.2% [w/v], incubation time (144 hour), and solid substrate (wheat straw) were the best conditions determined by the design of experiments using the Taguchi approach.

**Conclusion:**

Our finding showed that the use of rice straw and peanut pod, as solid-state substrates, led to 2.041-folds increase in the production of the enzyme, as compared to rice straw. In addition, the presence of an inducer did not have any significant impact on the enzyme production levels.

## INTRODUCTION

β -galactosidase (E.C 3.2.1.23), so called β-gal, is an important enzyme used in dairy industry and found abundantly in biological systems and microorganisms[1]. β-gal hydrolyzes the lactose (milk sugar) to its components, galactose and glucose. The enzyme is used for the improvement of milk and its derivatives for consumption by people with lactose intolerance, for prevention of lactose crystallization in frozen and condensed milk products and also for the increase of the sweetening properties of lactose[2,3]. Since a large number of people in different countries of the world[4] suffer from lactose intolerance, this matter makes the enzyme even more important.

In addition to health area, difficulties with lactose falls within two other main fields: food technology and environment[5]. From the food technology point of view, β-gal catalyzes transgalactosylation reaction, and lactose serves as a galactosyl donor and an acceptor to form di-, tri- or higher galactooligosaccharides[6,7]. Galactooligosaccharides are now considered as a probiotic food ingredient and have been demonstrated to promote the growth and the establishment of bifidobacteria in intestine[8,9], thus exerting a beneficial effect on the human host[10]. Until now, numerous studies concerning the production of galactooligosaccharides have been reported[11-15]. It has been indicated that galactooligosaccharides production from lactose is quite different in the final products and yields depending on the source of β-gal[15]. Lactose is associated with the high biochemical and chemical oxygen demand (BOD and COD) content of whey. β-gal is used commercially in lactose hydrolysis[16], which is an important application of this enzyme in food industry.

β-gal is found in nature and is able to release a wide range of compounds. The filamentous fungus *Aspergillus niger* is capable of producing different extracellular enzymes and has previously been used by various researchers for enzyme production[17-19]. In the present study, we have tried to establish optimal conditions for the highest production of β-gal by *A. niger* ATCC 9142 using indigenous and inexpensive wastes under solid-state fermentation (SSF) conditions with the assist of statistical experimental designs.

## MATERIALS AND METHODS

### Strain and culture media

*A. niger* ATCC 9142 was obtained from Iranian Research Organization for Science and Technology (IROST). Materials used in this study were obtained from Merck Co., Germany.

### Culture conditions and fermentation process

For preparation of pre-culture, one loop of fungal spores from slant tubes of potato dextrose agar medium was inoculated in a pre-culture medium plate, and the plate was kept at 30°C for 48 h. The pre-culture medium consisted of a potato dextrose agar enriched by 1 g/l yeast extract and 1 g/l glucose. In fact, yeast extract and glucose were implemented as the supplementation of nitrogen and carbon sources to improve the growth of fungi. Rice and wheat straw with different percentages of peanut pod were used as solid substrates for SSF. Mixed substrates (10 g) transferred to 500-ml Erlenmeyer flasks was moistened with the mineral medium [2.0 g (NH_4_)_2_SO_4_, 5.0 g KH_2_PO_4_ and 0.4 g MgSO_4_.7H_2_O per liter] to reach the final moisture content of 70% (w/v). After sterilization, the flasks were inoculated with pre-culture under sterile conditions. The flasks were then incubated at 30±1ºC. After the incubation period of fermentation, extraction of the enzyme was carried out, and the supernatant was used for analytical assays.

### Experimental design and analytical methods

The Taguchi method applies fractional factorial experimental designs, called orthogonal arrays, to reduce the number of experiments while obtaining statistically meaningful and worthwhile results. The selection of a suitable orthogonal array depends on the number of control parameters and their levels. An experimental L16 array from the Taguchi method was applied for the optimization of the enzyme production[5]. Five factors were selected: peanut pod (0, 30, 70 and 95% w/w), carbon/nitrogen (C/N) ratio (0.1, 1, 2 and 3% w/v), incubation time (69, 120, 144 and 168 h), type of solid substrate (wheat straw and rice straw) and lactose concentration (0 and 5% w/v). Inner arrays employed to assign the considered factors are shown in Table 1. For each experimental trial of the independent variables in the experimental design, the dependent parameter (enzyme production) was determined. The enzyme activity is shown in Table 2. A fractional factorial design with 16 different choice sets was used. Three factors at 4 levels and 2 factors at 2 levels were also examined. Normally, in the classical combination method using full factorial experimentation would require 4^3^×2^2^=256 experiments to capture the effective parameters[5]. Analysis of variance (ANOVA) was generated, and the effect of terms was determined (Table 3). The significance of all terms was judged statistically by computing the *P*<0.05. The data analyses and optimization process were generated using Minitab statistical software version 15[5].

**Table 1:** Assignment of experimental factors in Taguchi’s optimization method

Experiment number	C1	C2	C3	C4	C5
	A	B	C	D	E
1	1	1	1	1	1
2	1	2	2	1	1
3	1	3	3	2	2
4	1	4	4	2	2
5	2	1	2	2	2
6	2	2	1	2	2
7	2	3	4	1	1
8	2	4	3	1	1
9	3	1	3	1	2
10	3	2	4	1	2
11	3	3	1	2	1
12	3	4	2	2	1
13	4	1	4	2	1
14	4	2	3	2	1
15	4	3	2	1	2
16	4	4	1	1	2

C, column; A, peanut pod; B, C/N ratio; C, incubation time; D, solid substrate; E, lactose

**Table 2:** Experimental L16 orthogonal array and results of the β-galactosidase activity

Experiment number	Factor levels					Enzyme activity (U/mg)

	Peanut pod	C/N ratio	Incubation time	Solid substrate	Lactose concentration	
1	0	0.1	96	WS	0	8809
2	0	1.0	120	WS	0	3942
3	0	2.0	144	RS	5	2689
4	0	3.0	168	RS	5	82
5	30	0.1	120	RS	5	3613
6	30	1.0	96	RS	5	451
7	30	2.0	168	WS	0	4353
8	30	3.0	144	WS	0	4681
9	70	0.1	144	WS	5	3757
10	70	1.0	168	WS	5	2587
11	70	2.0	96	RS	0	4209
12	70	3.0	120	RS	0	1909
13	95	0.1	168	RS	0	1498
14	95	1.0	144	RS	0	2915
15	95	2.0	120	WS	5	1067
16	95	3.0	96	WS	5	164

WS, wheat straw; RS, rice straw

**Table 3:** Analysis of variance (ANOVA) of the regression parameters for the Taguchi method

Source	DF	Seq. SS	Adj. SS	Adj. MS	*F* value	*P* value
Peanut pod	3	13897678	13897678	4632559	2.14	0.238
C/N	3	15836903	15836903	5278968	2.43	0.205
Time (day)	3	5451074	5451074	1817025	0.84	0.539
Solid state	1	8655364	8655364	8655364	3.99	0.116
Lactose	1	19536400	19536400	19536400	9.01	0.040
Residual error	4	8675465	8675465	2168866	
Total	15	72052884		

DF, the degrees of freedom; Seq. SS, sequential sums of squares; Adj. SS, adjusted sums of squares; Adj. MS, adjusted mean square

### Enzyme assay

**Fig 1:**
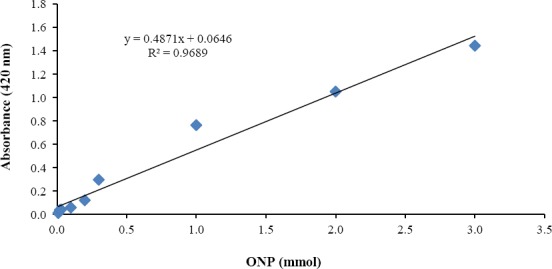
Standard calibration curve of o-nitrophenol (ONP)

The crude enzyme solution was diluted to a final volume of 0.5 ml with 0.1 M potassium phosphate buffer (pH 7.0) and then was added to 0.5 ml of 6 mM ortho-Nitrophenyl-β-D-galactopyranoside in the same buffer. The reaction mixture was incubated at 30°C for 20 min. The reaction was ended by adding 2 ml 0.1 M Na_2_CO_3_. β-gal assay was carried out spectrophotometrically. The enzyme activity was determined according to the slope of a standard calibration curve of ortho-nitrophenol (Fig. 1). The favorable regression of equation is indicative the accuracy of calculations and measurements. The concentration of ortho-nitrophenol released from ortho-Nitrophenyl-β-D-galactopyranoside was determined by measuring the absorbance at 420 nm. The enzyme activity was expressed as specific activity (U/mg), and one unit of β-gal activity (U) was defined as the amount of enzyme that liberates 1 µmol of ortho-nitrophenol per minute.

## RESULTS

**Fig 2:**
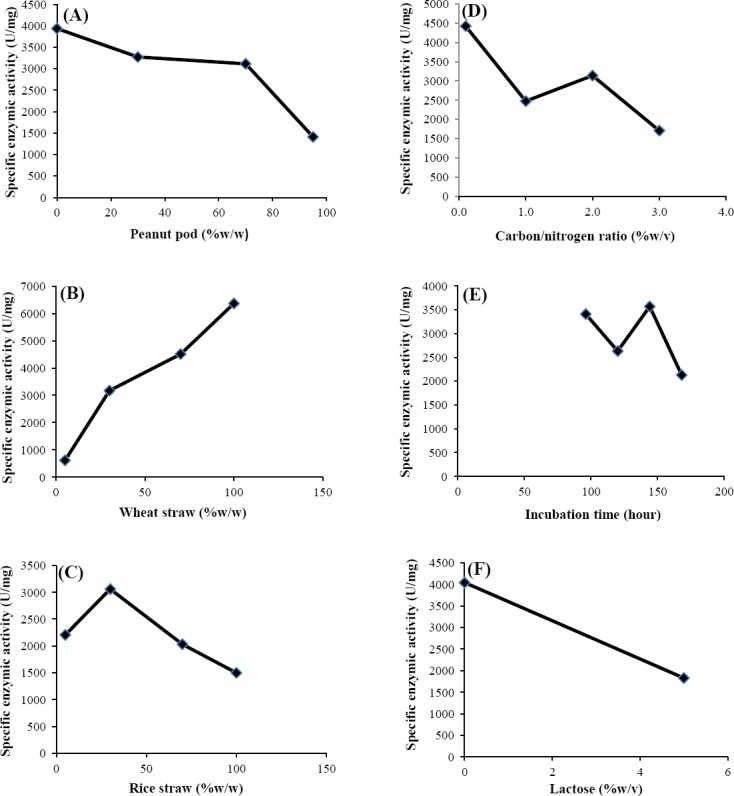
Effect of (A) peanut pod, (B) wheat straw, (C) rice straw, (D) carbon/nitrogen ratio, (E) incubation time, (F) lactose (as inducer) on the enzyme activity.

The influence of various solid-state substrates i.e. peanut pod, wheat straw and rice straw on the production of β-gal by *A. niger* ATCC 9142 are shown in Figures 2A-2C. The solid substrates screening showed that the wheat straw was the most suitable substrate for the production of the enzyme. Furthermore, the effects of different C/N ratios, the incubation time and different concentrations of lactose (as inducer) on the enzyme activity are indicated in Figures 2D-F.

Considering the various applied C/N ratios, the maximum enzyme production yield was found to be at 0.2% (w/v) (Fig. 2D). The optimum incubation time for enzyme activity in the crude extract was 144 h (Fig. 2E).

The effect of different concentration of lactose (0 and 5% w/v), as an inducer, on the β-gal production was also studied. The results showed that the addition of lactose to the basal medium did not have any significant impact on the enzyme production (Fig. 2F).

**Fig. 3:**
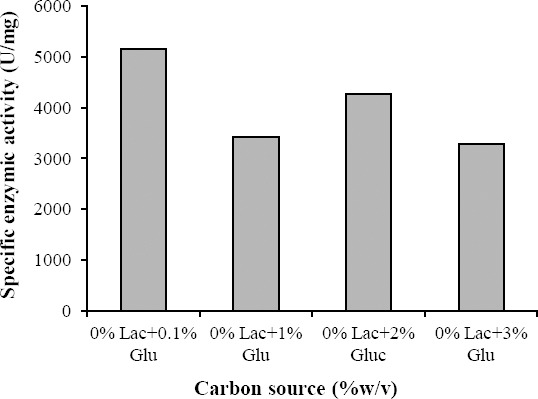
The effect of different carbohydrates on the induction of β-galactosidase in *A. niger*. Lac, lactose; Glu, glocuse

**Fig. 4:**
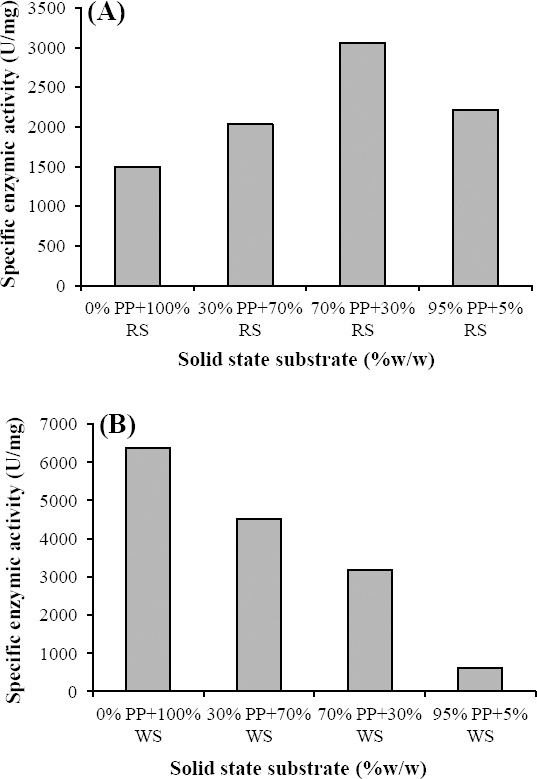
Enzyme activity of β-galactosidase using mixed substrates (A) peanut pod (PP) with rice straw (RS), (B) peanut pod with wheat straw (WS).

The enzyme activity exposed to different carbohydrates and mixed substrates ratios is demonstrated in Figures 3 and 4. As shown in Figure 3, glucose (0.1-%w/v) had the highest effect on the induction of β-gal.

The combination of rice straw and peanut pod led to an increasing in β-gal activity (approximately two-fold), as compared to the use of rice straw individually (Fig. 4A). However, for enzyme production, the use of wheat straw alone was more suitable than its combination with peanut pod (Fig. 4B).

**Fig.5:**
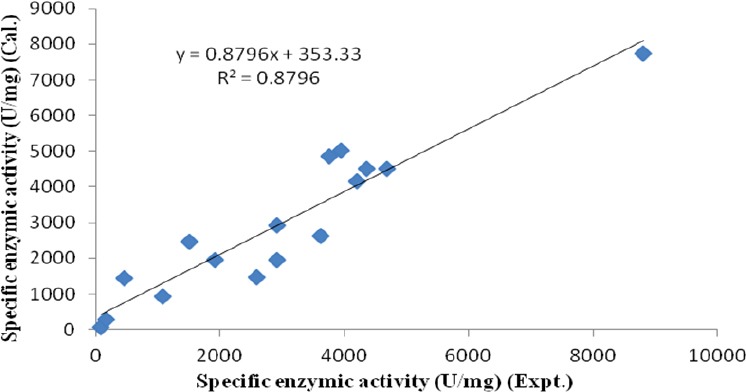
Relationship between the calculated yield of β-galactosidase and experimental data. Cal, calculated; Expt., experimental

The closer of R^2^ value to unity, the better the empirical models fits the actual data[20]. By the analysis of variance, the R^2^ value of this model was determined to be 0.879. Therefore, the developed model could adequately represent the real relationship among the parameters chosen (Fig. 5).

The experimental conditions for each run are included in Table 1. The orthogonal array designs of experimental parameters and the enzyme activity for each trail are shown in Table 2. As it is shown in Table 3, the analysis of variance (ANOVA) of the regression parameters for the Taguchi experimental design was evaluated. Response table for means is shown for parameters in Table 4.

**Table 4:** Response table for means

Level	Peanut pod	C/N	Time (day)	Solid state	Lactose
1	3937	4419	3408	3670	4040
2	3275	2474	2633	2199	1830
3	3116	3136	3567	
4	1411	1709	2130	
Delta	2526	2710	1437	1471	2210
Rank	2	1	5	4	3

## DISCUSSION

β-gal is one of the most important enzymes used in food processing and offers nutritional, technological and environmental applications. In the present study, the production of β-gal by SSF was measured and affected by a variety of physicochemical factors. The results indicated that all solid-state substrates could be used as suitable and low-cost substrates for β-gal production. The solid substrate screening showed that wheat straw was the most appropriate substrate for the growth of *A. niger* ATCC 9142. The highest β-gal activity was obtained at 8809 U/mg using wheat straw. Nizamuddin *et al*.[21] reported that wheat bran and rice husk support the maximal growth and β-gal production by *A. oryzae*. We found that the combination of 30% (w/w) rice straw with 70% (w/w) peanut pod led to ca. two-fold increase in supernatant β-gal activity (Fig. 4A). However, the combination of peanut pod and wheat straw resulted in dry culture, subsequent decrease in microbial growth and lower production level of β-gal.

The substrate level or water content is a critical factor in SSF. Therefore, an increase in substrate concentration or a decrease in water level leads to reduced solubility and minimizes heat exchange, oxygen transfer and low availability of nutrients to the culture, which affects microbial activity and results in decreased productivity[5].

The results of this study are useful to demonstrate the importance of solid-state fermentation for the production of β-gal using cost-free or low-cost agricultural by-products, which can be used as substrate(s).

The solid substrates used in this study offer significant benefits due to cheaper cost and abundant availability and the potential for production of high levels of enzyme.

Low and high amounts of carbohydrates can reduce yield of production[22]. A suitable concentration of carbohydrates raises the efficiency of production. Hence, investigations have been carried out to reveal the effect of various concentrations of glucose and yeast extract (0.1-3% [w/v]), as C/N sources, on the enzyme activity. Glucose and yeast extract were used as C/N sources, respectively. Numerous investigators have reported the carbon source regulation of β-gal biosynthesis in various microorganisms[23-28]. All of these studies have indicated that the role of carbon source in the biosynthesis of β-gal may vary and depend on the microorganisms used. Siddique *et al*.[29] have enhanced the production of β-gal using five organic nitrogen i.e. ammonium sulfate, corn steep liquor, diammonium phosphate, fish meal and urea with wheat bran, as a substrate, under SSF by *A. niger*. The highest level of β-gal activity was produced with corn steep liquor.

However, the effectiveness of the supplementation of these nutrients for the production of β-gal has not been studied to a great extent. Supplementation of nitrogenous sources especially yeast extract increases the amount of nutrients available to the bacteria, which could explain why there was an increase in the viable population of the organisms. This elevation may be attributed to the growth factors in addition to the nitrogen compounds present in yeast extract[30,31]. In experiments performed by Nizamuddin *et al*.[21], the optimization of β-gal was carried out in SSF. The fungal culture utilized several carbon sources for the β-gal induction. Glucose serves as the most suitable carbon source, followed by lactose, maltose and sucrose. Among the various nitrogen sources, sodium nitrate has been found to be the most promising[15]. Our results indicated that optimum ratio of C/N for enzyme activity in the crude extract was 0.1 % (w/v) (Fig. 2D). The culture with added lactose had a relatively constant decrease in enzyme induction in the presence of glucose (Fig. 3). This behavior can be expected in the presence of glucose and can be explained by the mechanism of catabolite repression, which will slow the rate of induction for a while[31]. Catabolic repression is the positive control of transcription. Glucose inhibits the synthesis of some inducible enzymes even in the presence of an inducer. This phenomenon is called catabolic repression or glucose effect and causes through the inhibition of cAMP synthesis. The cAMP is required for the initiation of transcription of many inducible enzymes[32]. Catabolite repression is one of the two known regulatory systems affecting the lac operon in the presence of glucose. It has previously been shown that the production of the lac operon enzymes can be induced to a high level using lactose analog in place of lactose while growing in a media devoid of any sugars. However, by the addition of glucose, the rate of induction would be severely repressed[33].

In the present investigation, the effect of incubation time of fermentation medium for enzyme production was studied in different time periods 96, 120, 144 and 168 hours. Incubation period is one of the major factors in cost effective production. Basil[34] observed that the addition of certain agricultural by-products (molasses and whey) to growth medium can enhance lactase activity. Similarly, Eratt *et al*.[35] and Sumitra *et al*.[36] reported that lactose and maltose improve the α-amylase activity on rice bran medium produced by *A. oryzae*. In contrast, Srinivas *et al*.[37] found a negative effect up to three days of fermentation and then turned positive with the presence of lactose, as a carbon source, for α-gal production by *A. niger*. Based on our results, the highest enzyme activity was observed at 144 h, and the enzyme activity indicated a periodic fluctuation over time (Fig. 2E). This phenomenon might be due to a change in the pH of the medium or denaturation and/or decomposition of β-gal as a result of interactions with other compounds in the fermented medium[5]. Similar findings have also been reported by Khayati *et al*. [5], Sandhya *et al*.[38] and Gupta *et al*.[39].

In conclusion, solid-state fermentation is well suited for the production of β-gal because of its abundant and cost-effectiveness.
